# Blood T1* correction increases accuracy of extracellular volume measurements using 3T cardiovascular magnetic resonance: Comparison of T1 and T1* maps

**DOI:** 10.1038/s41598-018-21696-0

**Published:** 2018-02-20

**Authors:** Yongning Shang, Xiaochun Zhang, Xiaoyue Zhou, Andreas Greiser, Zhengwei Zhou, Debiao Li, Jian Wang

**Affiliations:** 1 Department of Radiology, Southwest Hospital, Third Military Medical University, Chongqing, China; 2 MR Collaboration, Siemens Healthcare Ltd., Shanghai, China; 3 Siemens Healthcare GmbH, Erlangen, Germany; 4 Biomedical Imaging Research Institute, Cedars-Sinai Medical Center, Los Angeles, California, USA; 5 Department of Bioengineering, University of California Los Angeles, Los Angeles, California, USA

## Abstract

The goals were to compare the differences between ECV_L_ (extracellular volume derived from myocardial T1 and blood T1), ECV_c_ (combination of myocardial T1 and blood T1*), and ECVnL (derived from myocardium T1* and blood T1*), and to explore the diagnostic accuracy of these factors for discriminating between controls and patients. The Modified Look-Locker Inversion Recovery sequence was performed in 42 subjects to generate both T1 and T1* maps. Native and post-contrast T1 values for myocardium and blood pool were obtained, and ECVL, ECVc, and ECVnL were then calculated. The global ECVc values were smaller than the ECVL values (0.006, 2.11%, p < 0.001) and larger than the ECVnL values (0.06, 21.6%, p < 0.001) in all participants. The ECVc led to a 4–6% increase in the AUC value and a 24–32% reduction in the sample size to differentiate between the controls and other patients when compared with the ECVL. Blood T1* correction can improve the precision of blood T1 values and can consequently increase the accuracy of the extracellular volume fraction measurement. The ECVc can be used to improve diagnostic accuracy and reduce the sample size required for a clinical study.

## Introduction

Cardiac T1 mapping allows the quantitative measurement of myocardial and blood longitudinal relaxation T1^1^. The extracellular volume fraction (ECV), derived from native and post-contrast T1 mapping, reflects the size of the extracellular space in the myocardium and can be used as an important diagnostic biomarker of disease^2^, as well as for observation of disease progression^3^ and prognosis^4,5^. T1 mapping, together with the ECV, introduced a new frontier in radiology and cardiology, enabling the quantification of important tissue properties of both local and global myocardium, independent of function^6^.

T1 mapping can be performed utilizing several techniques, such as Saturation recovery Single-sHot Acquisition (SASHA)^7^, saturation pulse prepared heart-rate-independent inversion recovery (SAPPHIRE)^8^, and a widely used ECG-triggered method (Modified Look-Locker Inversion Recovery, MOLLI)^9,10^. Although the effects of T2 sensitivity, magnetization transfer, and inversion efficiency lead to an underestimation of myocardial T1 values^11^, the higher signal-to-noise ratio (SNR), which is due to IR preparation and a large number of images, permits MOLLI to more precisely measure T1 values than SASHA. Compared with SASHA and its modified version (SAPPHIRE), MOLLI and the shortened MOLLI (ShMOLLI)^12^ yield higher precision and lower accuracy^11,13^. Modifications of MOLLI sampling schemes (such as 5(3)3 and 4(1)3(1)2 for native and post-contrast T1 mapping^14^) have been proposed to reduce breath-hold duration and reduce heart rate sensitivity, which incrementally increase the precision of T1 measurements. To summarize, MOLLI has been widely accepted and used for the precise measurement of myocardial T1.

Calculation of the ECV is based on the measurement of both native and post-contrast T1 of the myocardium and the blood pool and is calibrated using the hematocrit (HCT) value^15^. In previous studies, the ECV was obtained by manually drawing regions of interest (ROIs) on T1 maps. The application of the motion correction (MOCO) technique can eliminate the effect of respiratory and heart motion to allow the calculation of a more accurate myocardial T1 value and permit the generation of fully automated calculated pixel-wise ECV maps^16,17^. However, due to the inflow effect of the blood pool, the T1 values of the blood pool and the myocardium differ in the measurement. More specifically, for the measurement of blood T1, because there is inflow or replacement of fresh blood with each heartbeat, the Look-Locker (LL) correction should be eliminated to provide a more realistic estimation of the blood T1.

ECV measurements based on the blood T1 values from LL-generated maps may lead to misestimation of their true values, which will likely limit the clinical implications of ECV in disease diagnosis, observation of disease progression, and prognosis. Theoretically, T1* maps, based on image registration without the LL correction, can therefore be used for more accurate measurement of blood T1 values, and thus, a more accurate ECV.In a previous study^18^, Zhao *et al*. described the LV ECV calculated from myocardial T1 and blood T1* in patients with atrial fibrillation but did not compare this measurement method with others. Nickander *et al*. reported that blood T1 and T1* correction reduced the variability in native myocardial T1 values^19^. They did not calculate ECV, but their results placed much importance on the effects of blood values in the myocardium. Considered together, blood T1* values should be prioritized to obtain more accurate measurements of the ECV.

Therefore, we hypothesized the following: 1) There may be differences in blood T1 values obtained from T1 and T1* mapping; 2) ECV can be more accurately calculated from myocardial T1 and blood T1*; and 3) The more precise ECV can improve the accuracy of disease diagnosis and reduce the sample size needed to identify the difference in the ECV between the controls and patients.

## Results

The demographic and left ventricular functional parameters of all the participants are shown in Table 1.

**Table 1:** Demographic and left ventricular functional parameters of all the participants.

Parameters	All participants (n = 42)
Age, years	55.0 ± 10.6
Gender, male/female	25/17
Height, m	1.62 ± 0.09
Weight, kg	63.9 ± 9.5
Body mass index, kg/m^2^	24.2 ± 2.7
Body surface area, m^2^	1.68 ± 0.16
Systolic blood pressure, mmHg	119.4 ± 13.8
Diastolic blood pressure, mmHg	77.6 ± 9.4
Heart rate, bpm	73.6 ± 11.0
Ejection fraction, %	58.5 ± 7.3
End-diastolic volume index, ml/m^2^	70.4 ± 12.7
End-systolic volume index, ml/m^2^	29.5 ± 10.1
Stroke volume index, ml/m^2^	40.9 ± 7.6
Cardiac index, l/min/m^2^	2.99 ± 0.59
Myocardial mass index, g/m^2^	57.9 ± 13.4
Hematocrit, %	39.8 ± 3.4
Late gadolinium enhancement positive, n (%)	7 (16.7)

Data were presented as mean ± standard deviation.

Total number: type 2 diabetes mellitus (T2DM) without hypertension (HT) (n = 19), T2DM with HT (n = 5), HT (n = 2), healthy volunteers (n = 7), hypertrophic cardiomyopathy (n = 4), cardiac amyloidosis (n = 1), chronic myocardial infarction (n = 1), left ventricular noncompaction (n = 1), dilated cardiomyopathy (n = 1), arrhythmogenic right ventricular cardiomyopathy (n = 1).

### Comparison of native and post-contrast T1 values of the myocardium and blood between T1 and T1* mapping in all participants

**Figure 1:**
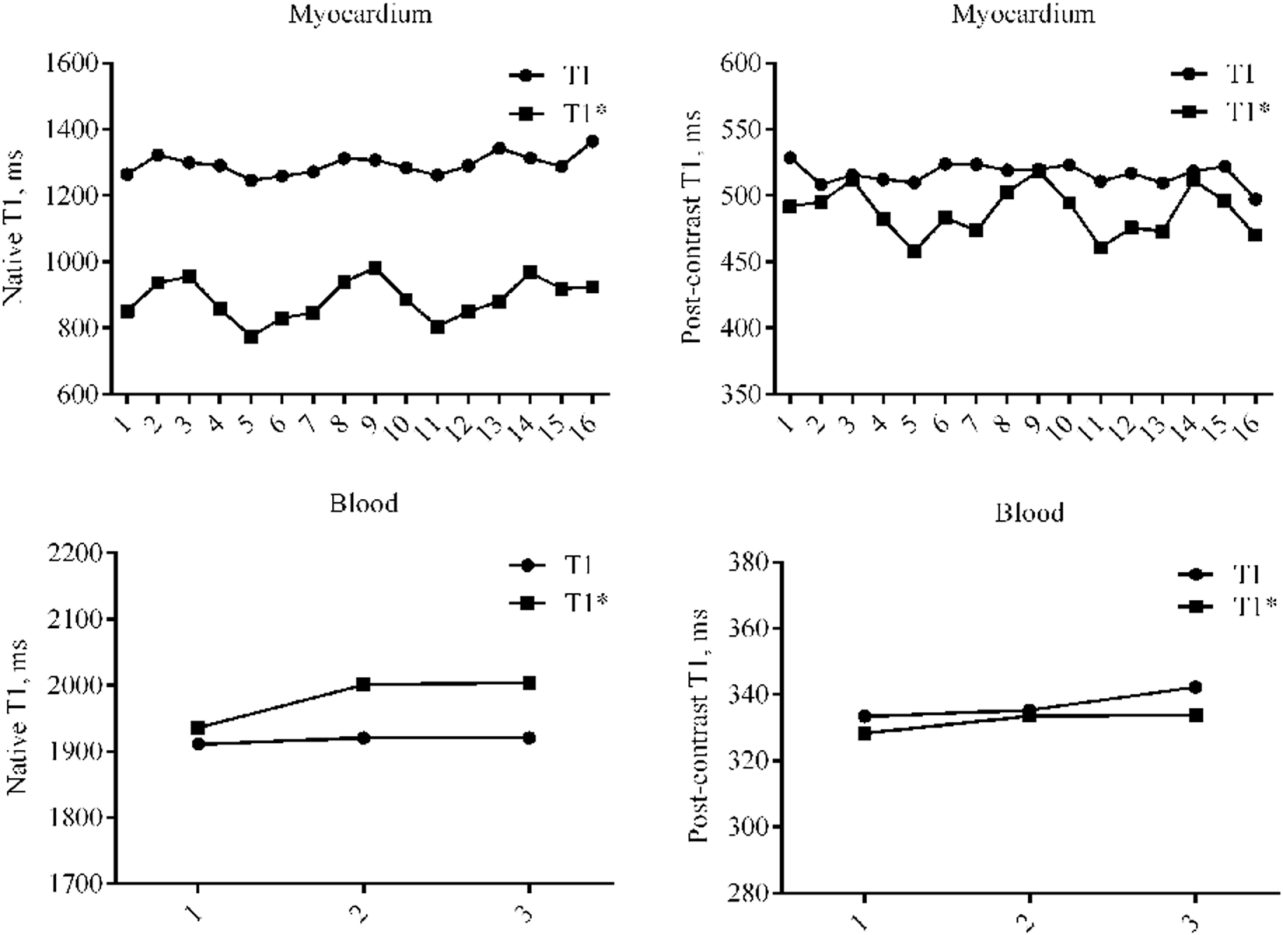
Superimposed symbols with connecting line of T1 and T1* values. Superimposed symbols with connecting line showing native and post-contrast T1 and T1* maps of American Heart Association 16-segment myocardium and three slices blood in all subjects.

Both the native and post-contrast T1 values of the sixteen segments of the LV myocardium and blood pools in the three slices are presented in Fig. 1. In addition, Supplementary Figure S1 shows the native and post-contrast T1 values from the T1/T1* mapping of the LV global myocardium and blood pool. There were significant differences in myocardial and blood T1 values between the T1 and T1* maps (based on paired t-tests) for myocardial native (p < 0.001), post-contrast T1 (p < 0.001), blood native (p = 0.003), and post-contrast T1 values (p < 0.001).

### Comparison of LV myocardial ECV_L_ and ECVc in all participants

**Figure 2:**
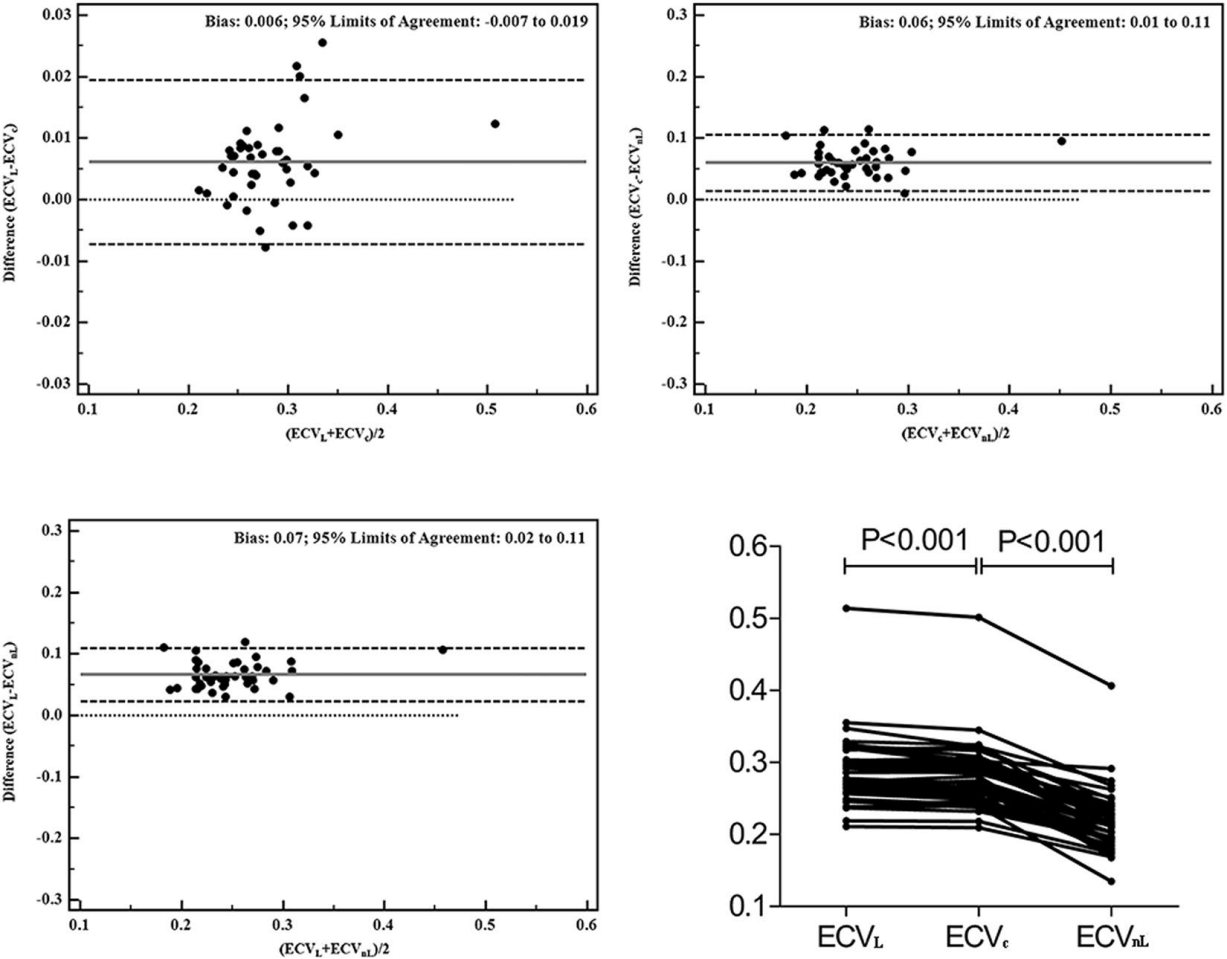
Bland-Altman plots of ECV. Bland-Altman plots and superimposed symbols with connecting line show comparisons among the global ECVL, ECVc, and ECVnL.

The sixteen segments and the ECVL (derived from myocardial T1 and blood T1) and ECVc (combination of myocardial T1 and blood T1*) of the global LV myocardium in all the participants are shown in Fig. 2 and supplementary Figure S2, and the results are summarized in Table 2. The global ECVL was strongly correlated with the ECVc (correlation coefficient = 0.991, p < 0.001). The mean difference between the global ECVL and ECVc was 0.006 (95% confidence interval, −0.007 to 0.019), and the global ECVL was 2.11% larger than the global ECVc (p < 0.001). In addition, the sixteen segments and the ECVL were also consistently correlated with ECVc (all the correlation coefficients were > 0.9, all p < 0.001). Paired t-tests and the Bland-Altman plots showed that the ECVL was larger than the ECVc (mean differences ranged from 0.004 to 0.011); these were statistically significant (all p < 0.01), though the mean differences were small.

**Table 2:** Comparison of LV myocardial ECVL and ECVc in all participants.

AHA Segments	ECVL	ECVc	ECVL − ECVc	(ECVL − ECVc)/ ECVL), %	Correlation coefficient	P
1	0.266 ± 0.050	0.261 ± 0.049	0.005	1.88	0.988*	<0.001
2	0.293 ± 0.051	0.287 ± 0.049	0.006	2.04	0.986*	<0.001
3	0.284 ± 0.055	0.278 ± 0.053	0.006	2.11	0.989*	<0.001
4	0.287 ± 0.068	0.281 ± 0.066	0.006	2.09	0.992*	<0.001
5	0.282 ± 0.071	0.277 ± 0.069	0.006	2.13	0.993*	<0.001
6	0.269 ± 0.050	0.263 ± 0.049	0.005	1.86	0.988*	<0.001
7	0.273 ± 0.045	0.269 ± 0.044	0.004	1.46	0.981*	0.010
8	0.283 ± 0.047	0.279 ± 0.046	0.004	1.41	0.982*	0.009
9	0.282 ± 0.050	0.278 ± 0.049	0.004	1.42	0.984*	0.010
10	0.276 ± 0.055	0.272 ± 0.054	0.004	1.45	0.988*	0.008
11	0.283 ± 0.055	0.280 ± 0.054	0.004	1.41	0.987*	0.010
12	0.281 ± 0.046	0.277 ± 0.044	0.004	1.42	0.981*	0.008
13	0.304 ± 0.054	0.293 ± 0.055	0.010	3.29	0.953*	<0.001
14	0.290 ± 0.046	0.280 ± 0.044	0.010	3.45	0.941*	<0.001
15	0.284 ± 0.051	0.274 ± 0.048	0.010	3.52	0.955*	<0.001
16	0.318 ± 0.049	0.306 ± 0.045	0.011	3.46	0.936*	<0.001
Global	0.285 ± 0.049	0.278 ± 0.047	0.006	2.11	0.991*	<0.001

Correlation coefficient: linear regression between ECVL and ECVc, *all p < 0.001

AHA: American Heart Association.

### Comparison of the LV myocardial ECVc and ECVnL in all participants

The ECVc and ECVnL (derived from myocardium T1* and blood T1*)of the global LV myocardium and sixteen segments in all the participants are shown in Fig. 2 and supplementary Figure S2 and are summarized in Table 3. The global ECVc was strongly correlated with the ECVnL (correlation coefficient = 0.871, p < 0.001). The global ECVc was 21.6% larger than the global ECVnL (p < 0.001), and the mean difference was 0.06 (95% confidence interval, 0.01 to 0.11). The ECVc of the sixteen segments was also correlated with the ECVnL (correlation coefficient ranged from 0.539 to 0.908, all p < 0.001). Moreover, all the ECVc values were significantly larger than the ECVnL (mean differences ranged from 0.050 to 0.070, all p < 0.001).

**Table 3:** Comparison of LV myocardial ECVc and ECVnL in all participants

AHA Segments	ECVc	ECVnL	ECVc − ECVnL	(ECVc − ECVnL)/ ECVc), %	Correlation coefficient	P
1	0.261 ± 0.049	0.206 ± 0.055	0.055	21.1	0.826*	<0.001
2	0.287 ± 0.049	0.226 ± 0.049	0.061	21.3	0.821*	<0.001
3	0.278 ± 0.053	0.215 ± 0.052	0.063	22.7	0.785*	<0.001
4	0.281 ± 0.066	0.211 ± 0.074	0.070	24.9	0.723*	<0.001
5	0.277 ± 0.069	0.210 ± 0.074	0.067	24.2	0.728*	<0.001
6	0.263 ± 0.049	0.207 ± 0.066	0.057	21.7	0.861*	<0.001
7	0.269 ± 0.044	0.219 ± 0.047	0.050	18.6	0.815*	<0.001
8	0.279 ± 0.046	0.220 ± 0.041	0.059	21.1	0.812*	<0.001
9	0.278 ± 0.049	0.217 ± 0.042	0.061	21.9	0.908*	<0.001
10	0.272 ± 0.054	0.211 ± 0.053	0.061	22.4	0.818*	<0.001
11	0.280 ± 0.054	0.216 ± 0.054	0.064	22.9	0.678*	<0.001
12	0.277 ± 0.044	0.219 ± 0.042	0.058	20.9	0.864*	<0.001
13	0.293 ± 0.055	0.230 ± 0.048	0.063	21.5	0.539*	<0.001
14	0.280 ± 0.044	0.221 ± 0.043	0.059	21.1	0.711*	<0.001
15	0.274 ± 0.048	0.220 ± 0.043	0.054	19.7	0.695*	<0.001
16	0.306 ± 0.045	0.245 ± 0.052	0.062	20.3	0.668*	<0.001
Global	0.278 ± 0.047	0.218 ± 0.044	0.060	21.6	0.871*	<0.001

Correlation coefficient: linear regression between ECVc and ECVnL, *all p < 0.001

AHA: American Heart Association.

Regarding the comparison between the ECVL and ECVnL, all the data are summarized in Supplementary Table S1.

### Sample size using the ECV_L_ and ECV_c_ for identifying the differences in ECV between the controls and patients

The mean and standard deviation (SD) of the ECVL and ECVc are summarized in Table 4. The SD of the ECVc was smaller than that of the ECVL in the T2DMs without HT, controls, and cardiomyopathies. Regarding the mean difference between the T2DMs without HT and controls, the ΔECVc was 3% larger than the ΔECVL, whereas for the mean difference between the cardiomyopathies and controls, the ΔECVc was 19% larger than the ΔECVL. As a result, the reduction in SD and increase in the mean difference reduced the total sample size for identifying the differences between the T2DMs patients without HT and the controls from 63 to 48 (24% reduction in sample size). These factors reduced the total sample size for identifying p < 0.001 the differences between the cardiomyopathies and controls from 62 to 42 (32% reduction in sample size).

**Table 4:** The ECV_L_ and ECV_c_ of the T2DMs patients without HT, controls, and cardiomyopathies

	T2DMs without HT (n = 19)		Controls (n = 7)		Cardiomyopathies (n = 9)	
	Mean	SD	Mean	SD	Mean	SD
ECV_L_	0.2860	**0.0368**	0.2672	**0.0155**	0.3086	**0.0834**
ECV_c_	0.2786	**0.0333**	0.2593	**0.0138**	0.3086	**0.0793**
	T2DMs without HT - Controls			Cardiomyopathies - controls		
ΔECV_L_	0.0188			0.0414		
ΔECV_c_	0.0193			0.0493		
ΔECV_c_–ΔECV_L_	**0.0005**			**0.0079**		
(ΔECV_c_–ΔECV_L_)/ΔECV_L_	**3%**			**19%**		

SD: standard deviation, T2DMs without HT: type 2 diabetes mellitus without hypertension.

### Comparison of the diagnostic accuracy of the ECV_L_ and ECV_c_ to discriminate between the controls and patients

**Figure 3:**
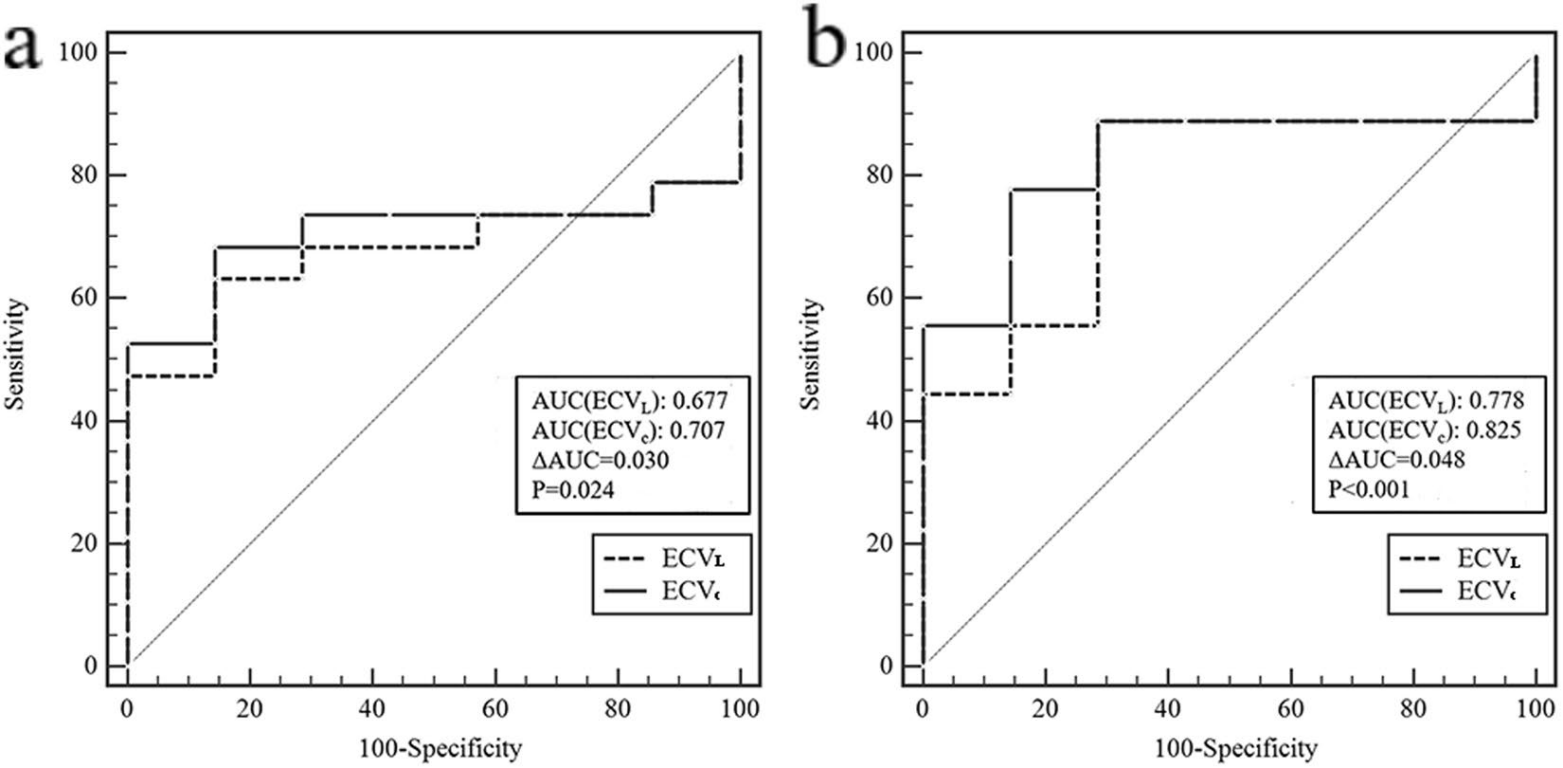
Receiver operating characteristic (ROC) curves. ROC curves showing the capacity of the ECVL and ECVc to discriminate between controls and others. (**a**) ROC curves show the capacity of the ECVL and ECVc to discriminate between controls and diabetes patients without hypertension. (**b**) ROC curves show the capacity of the ECVL and ECVc to discriminate between controls and patients with cardiomyopathies.

To differentiate between the controls and T2DM without HT, the AUC values for the ECVL and ECVc were 0.677 and 0.707, respectively, and the difference between the two AUC values was statistically significant (ΔAUC = 0.030, 4%, P = 0.024, Fig. 3(a). To differentiate between the controls and patients with cardiomyopathies, the AUC values for the ECVL and ECVc were 0.778 and 0.825, respectively, and the difference between the two AUC values was statistically significant (ΔAUC = 0.0480, 6%, P < 0.001, Fig. 3(b). Taken together, the ECVc can improve the diagnostic accuracy for discriminating between the controls and patients.

### Intra- and inter-observer reproducibility

Bland-Altman diagrams for the ECVL, ECVc, and ECVnL of the sixteen segments of the myocardium are shown in Supplementary Figure S3–8. Summaries of the Bland-Altman statistics and ICC values for the intra- and inter-observer differences are shown in Supplementary Tables S2–4.

## Discussion

There were significant differences in both the native and post-contrast T1 values of blood and myocardium obtained from the T1 and T1* maps. The ECVc was significantly smaller than the ECVL whereas it was larger than the ECVnL. In addition, the ECVc can reduce the total sample size needed for identifying differences in the ECV between the controls and patients, and the ECVc can improve the diagnostic accuracy for discriminating between the controls and patients.

We found that the blood T1* values were higher compared to the blood T1 values for the native T1 acquisitions and were mirrored by inverse effects in post-contrast acquisition in all the participants. Our results are consistent with those in a study conducted by Nickander^19^, where mean blood R1 (1/T1) was 0.00064 ± 0.00004 ms-1 and mean blood R1* (1/T1*) was 0.00061 ± 0.00005 ms-1 at 1.5 T CMR. Although the differences were not compared, those data showed a trend suggesting that the blood T1* value was larger than the blood T1 value. In MOLLI, due to the recurrent signal readout after a single inversion, the magnetization returns to steady state more rapidly than in free relaxation, and this is addressed by the Look-Locker correction. In this study, a true Look-Locker experiment with MOLLI was not performed, as there were long episodes of free relaxation disturbed by a few readout trains. Nevertheless, there was an “enforced” decay that could have been addressed by correction, as proposed for the original Look-Locker method. As the blood did not see the repeated readout pulse trains, it should not require this type of correction. Therefore, blood T1 value measurements are not sufficiently accurate on the T1 maps generated by the LL technique or its derived MOLLI prototype. T1*, as one of the three parameters in a three-parameter model to obtain T1 maps, is initialized as the linearly interpolated zero-crossing time generated from the “polarity” corrected signal intensity curve^20^. The pixel-wise T1* parametric maps, which have not undergone the LL correction, should be used for more accurate measurements of the blood T1 values^18^. Therefore, there is theoretically a significant difference in the blood T1 obtained from the T1 and T1* maps. Of note, our *in vivo* study results corroborate this theory. In future studies, T1* maps should be used for more accurately measuring the blood T1.

The inversion efficiency is another important factor that can influence the accuracy of MOLLI-based T1 estimation to a certain extent. Recent studies^21,22^ indicated that the inversion factor can be applied to correct T1 (T1corrected = T1/ inversion factor) to improve MOLLI T1 estimation accuracy. Although it can be estimated by simulating the adiabatic inversion pulse^21^ for a certain tissue, the actual *in vivo* inversion factor can be significantly different from the theoretical value. Further study^23^ showed that the actual *in vivo* inversion factor may be dependent on the MRI hardware and may need to be measured *in vivo* for each MRI scanner. Our present study focused on T1*, so the influence of inversion factor on T1 estimation was not explored. In view of its importance, inversion factor shall be attached to more attention in the future studies.

Besides the original three-parameter exponential curve fitting T1 estimation algorithm, several T1 estimation algorithms have recently been proposed for the MOLLI sequence, including the Bloch equation simulation with slice profile correction (BLESSPC) T1 estimation^24^, the inversion group (IG) fit^25^ and the Instantaneous Signal Loss simulation (InSiL) T1 estimation^23^. Shao *et al*.^22^ compared four T1 estimation algorithms for MOLLI with inversion factor correction and indicated that BLESSPC has superior accuracy and is the least sensitive to the flip angle, the heart rate and the acquisition scheme variations than the original fit, IG fit and InSiL, but the original fit has superior precision than the other three methods for T1 > 400 ms. Due to the absence of *in vivo* inversion factor, diagnostic accuracy for ECV of the “no correction” T1* approach was not compared with BLESSPC, IG fit and InSiL in the present study. It is interesting to see which method can provide better diagnostic accuracy for ECV in the future.

ECV was introduced to overcome the limitations of post-contrast T1 values in assessing diffuse abnormalities, one of which is the type and dose of contrast agent^26^, injection scheme^27^, or time point post-contrast image acquisition^28^, among other factors. The ECV is not sensitive to these effects, accounting for the blood T1 values and variation in the HCT^1^. Therefore, besides the myocardial issue, blood T1 values should also be seriously considered. We found that for the native T1 value of blood, T1* was higher than T1; for the post-contrast T1 value of blood, T1* was lower than T1. Thus, the ECVc is consequently lower than the ECVL.

Recently, much effort has been put to explore the feasibility of the synthetic ECV which derived from the blood T1 value and to validate its clinical value. Some studies^29,30^ has demonstrated that the synthetic ECV strongly correlates with the conventionally calculated ECV (all R^2^ > 0.90 at 1.5 T MR and 3.0 T MR) and can be an alternative to it without blood sampling. However, Raucci *et al*.^31^ reported that although it strongly correlates with conventionally calculated ECV (0.80 < R^2^ < 0.90 at 1.5 T MR), synthetic ECV can result in miscategorization of individual patients, especially in pediatric and young adult patients. Although the clinical value of synthetic ECV is still on the line, the accuracy of blood T1 values is being embraced as a very important factor in ECV calculation. The present study did not focus on estimating the synthetic ECV, but more attention will be attached to it in our future study.

Our results highlight the complexity of performing ECV measurements, but they also stress the importance of post-processing methods aimed at increasing accuracy and enhancing clinical diagnostic use. While some diseases such as MI or CAL show a significant increase in the ECV, more subtle differences are obtained in conditions with less myocardial damage, such as T2DM, HT, or HCM without LGE^32^. In Wong’s study^5^, which comprised 231 patients with diabetes and 945 participants without diabetes, the median ECV of the participants with and without diabetes were 0.302 and 0.281, respectively. The difference between the two groups was about 0.021 (7%). Although the difference in the ECV of the two groups was significant, there was a marked overlap of the ECV between the two groups. Nevertheless, the ECV was determined to be an important prognostic index for predicting mortality and/or incident hospitalization for heart failure in diabetes. Therefore, even small differences between the values calculated from unstandardized sequences or post-processing procedures may limit the clinical utility of the ECV. Notably, the accuracy and precision of the ECV measurements are thus far unclear because of the difficulty in obtaining *in vivo* reference values for ECV measurements. Previous studies have demonstrated that different reference values for the ECV were acquired in studies with different field strengths and sequences^11,33^. Regarding our results in the present study, there was a minor but statistically significant difference between the ECVL and ECVc measurements, which indicated an incremental role of blood T1 measurements accuracy in ECV values. Further studies are needed to confirm the reference values for the ECVc measurements in the normal controls and patients.

Our results indicate that the ECVc led to an increase in diagnostic accuracy compared with ECVL, which may be attributed to the ECVc-associated larger mean difference between the groups and smaller SD in each group. This might have significant clinical implications for diseases that are characterized by an alteration in the ECV, such as amyloidosis, cardiomyopathies, HT, and/or T2DM-related cardiac diseases. With increased diagnostic accuracy, these diseases could potentially be detected earlier when only subtle differences in the ECV may be present. In addition, the 4–6% increase in diagnostic accuracy means that 4–6% subjects can be clearly diagnosed and treated in time, which is very important for patients.

### Limitations

This study has several limitations. First, the sample size of this study was relatively small, more specifically the proportion of patients with serious myocardial damage (such as MI, myocarditis, HCM, CAL, etc.). Because the complexity of enrolled patients can test the robustness and efficiency of the ECV calculation method, more patients should be included in future studies. Second, considering that the ECVc measurements were derived from myocardium T1 maps and blood T1* maps, T1* maps cannot be generated using cvi42. Thus, although the ECV values can be directly obtained from ECV maps, ECVc values cannot be directly obtained from T1* maps. In this study, we acquired the T1 and T1* values of the myocardium and blood on native and post-contrast T1 and T1* maps and subsequently calculated the ECVL and ECVc. Although this was time-consuming and relatively complicated, the process can reduce errors substantially. In the future, the importance of ECVc values should be further investigated to directly generate T1* maps.

## Conclusions

Blood native T1* is longer than native T1 and post-contrast T1* is shorter than post-contrast T1. The ECVc is smaller than the ECVL and larger than the ECVnL. In general, blood T1* correction can improve the accuracy of blood T1 values and can consequently increase the accuracy of extracellular volume fraction measurement. The ECVc can be used to improve diagnostic accuracy and reduce the sample size required for clinical study.

## Methods

### Study population

All the recruited participants were scanned with cardiac magnetic resonance (CMR) between January and March 2017, in our hospital. Three patients who did not receive gadolinium injections were excluded, and therefore a total of 42 participants were included, comprising nineteen patients with type 2 diabetes mellitus (T2DMs) without hypertension (HT), five with T2DMs with HT, two patients with HT, four with hypertrophic cardiomyopathy (HCM), and one each with cardiac amyloidosis (CAL), chronic myocardial infarction (MI), left ventricular noncompaction (LVNC), dilated cardiomyopathy (DCM), and arrhythmogenic right ventricular cardiomyopathy (ARVC). Seven healthy volunteers, who were not referred as patients for normal manifestation on clinical CMR and without risk factors or evidence of cardiovascular disease, were also recruited. The study was approved by the Southwest Hospital Ethics Committee (reference number, 2016-Scientific-Research-No. 50) and was performed in accordance with the Declaration of Helsink and all the participants provided written informed consent.

### Cardiac Magnetic Resonance protocols

All CMR was performed using a 3 T MAGNETOM Trio a Tim System (Siemens Healthcare, Erlangen, Germany) with a 6-channel body matrix coil plus 2 rows of the spine array coil. A prototype for patient-specific, localized shimming in the heart was used to improve field uniformity. Blood samples were collected about 30 minutes before scanning and were immediately sent to the Department of Clinical Laboratory to obtain the HCT.

We compared the differences in T1 values of the myocardium and blood acquired from both T1 and T1* mapping; compared the differences in ECV_L_ (derived from LL-corrected myocardial T1 and blood T1), ECV_c_ (combination of LL-corrected myocardial T1 and non-LL-corrected blood T1*), and ECVnL (derived from non-LL-corrected myocardium T1* and blood T1*); calculated the sample size using the ECVL and ECVc for identifying differences in the ECV between the controls and patients; and compared the differences in the diagnostic accuracy of the ECVL and ECVc to discriminate between the controls and patients.

### Cine

Electrographic-gated, breath-hold steady-state free-precession (SSFP) short-axis retro-gated cine images covering the entire left ventricle (LV) were taken at 6 mm slice thickness, 1.5 mm slice gap, field of view (FOV) 325 × 400 mm², matrix 179 × 256, repetition time (TR) 59.22 ms, and echo time (TE) 1.45 ms, and at 25 phases per cardiac cycle. All the cine images were analyzed offline on a workstation with Argus software (Siemens Healthcare, Erlangen, Germany). Endo- and epicardial contours were manually traced to measure the LV cavity at end-diastole and end-systole. The papillary muscles were included in the LV volume. The LV end-diastolic volume index (EDVi), end-systolic volume index (ESVi), stroke volume index (SVi), ejection fraction (EF), cardiac index (CI), and myocardial mass index (MMi) were obtained.

### Late Gadolinium Enhancement (LGE)

Segmented LGE images covering the entire LV were performed using the Phase Sensitive Inversion Recovery (PSIR) sequence (TR/TE 680/1.94 ms, 8 mm slice thickness, 1.6 mm slice gap, FOV 325 × 400 mm²) approximately 10 min after a bolus administration of 0.2 mmol/kg gadodiamide (Omniscan, GE Healthcare).

### T1 mapping

A breath-hold ECG-gated, MOLLI prototype with a 5(3)3 and 4(1)3(1)2 sampling pattern was performed for native and post-contrast T1 mapping, respectively, with FOV 400 × 300 mm², matrix 256 × 166, and 6 mm thickness. Basal, mid-ventricular, and apical short-axis images were acquired before and approximately 15 min after the administration of gadodiamide. T1 maps were generated inline from the MOLLI images with MOCO and T1* maps were generated from the images without the Look-Locker correction. All the T1 and T1* maps were transferred to the cvi42 software (Circle Cardiovascular Imaging Inc., Calgary, Alberta, Canada) for offline analysis.

**Figure 4:**
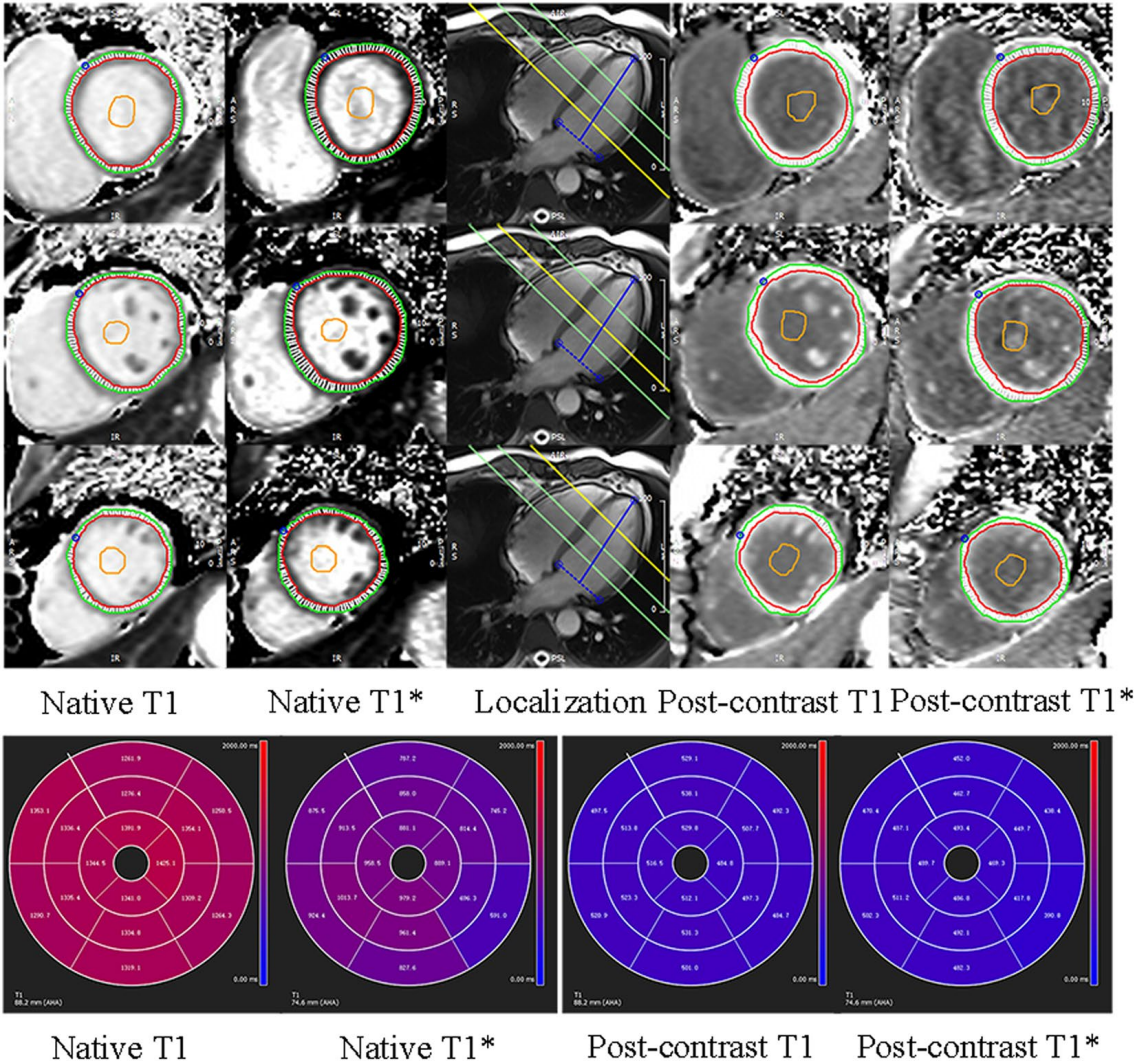
Short-axis T1 and T1* maps. Images show example of native and post-contrast T1 and T1* maps of three slices and the corresponding American Heart Association sixteen segments in one participant.

The LV endo- and epicardial borders were manually delineated with attention paid to avoiding partial-volume effects from the blood pool and epicardial fat. Sixteen myocardial segments (according to the American Heart Association, myocardial 17-segment classification with exclusion of the apical segment) and global native and post-contrast T1 and T1* values were obtained. The regions of interest (ROIs) were manually drawn with care in the LV cavity to avoid the papillary muscles and myocardium. Global and three-slices blood native and post-contrast T1 and T1* values were measured (Fig. 4). The global and 16-segmental myocardial ECV were calculated from native and post-contrast T1 and T1* maps using the following equations:

1 $$EC{V}_{L}=(1-{\rm{HCT}})\frac{\frac{1}{{\rm{T}}1\,{\rm{myo}}\,{\rm{post}}}-\,\frac{1}{{\rm{T}}1\,{\rm{myo}}\,{\rm{native}}}}{\frac{1}{{\rm{T}}1\,{\rm{blood}}\,{\rm{post}}}-\frac{1}{{\rm{T}}1\,{\rm{blood}}\,{\rm{native}}}}$$

2 $$EC{V}_{c}=(1-{\rm{HCT}})\frac{\frac{1}{{\rm{T}}1\,{\rm{myo}}\,{\rm{post}}}-\frac{1}{{\rm{T}}1\,{\rm{myo}}\,{\rm{native}}}}{\frac{1}{{{\rm{T}}}_{1}^{\ast }\,{\rm{blood}}\,{\rm{post}}}-\frac{1}{{{\rm{T}}}_{1}^{\ast }\,{\rm{blood}}\,{\rm{native}}}}$$

3 $$EC{V}_{nL}=(1-{\rm{HCT}})\frac{\frac{1}{{{\rm{T}}}_{1}^{\ast }\,{\rm{myo}}\,{\rm{post}}}-\frac{1}{{{\rm{T}}}_{1}^{\ast }\,{\rm{myo}}\,{\rm{native}}}\,}{\frac{1}{{{\rm{T}}}_{1}^{\ast }\,{\rm{blood}}\,{\rm{post}}}-\frac{1}{{{\rm{T}}}_{1}^{\ast }\,{\rm{blood}}\,{\rm{native}}}}$$

### Statistical analysis

The data were expressed as mean and standard deviation. Differences between the means for all the participants were compared using the paired t-test and the Bland-Altman method^34^. The relationships between bivariates were analyzed using Pearson’s method. For inter-observer reproducibility, images of ten randomly selected participants were independently analyzed by two radiologists (Shang Y and Zhang X, each with more than three years’ experience). For intra-observer reproducibility, one radiologist (Shang Y) reanalyzed images of ten participants after one month. The intra- and inter-observer variabilities for the ECV were visualized as Bland-Altman plots and analyzed by determining the intra-class correlation coefficient (ICC). The values for the area under the receiver operating characteristic (ROC) curves (AUC) were calculated to compare the capacity of the ECVL and ECVc to discriminate between the controls and others. The sample size was also calculated. Statistical tests were two-tailed, and the statistical significance was defined as P < 0.05. Statistical analysis was performed using SPSS (version 21.0, SPSS Inc., Chicago, IL, USA), GraphPad Prism (version 6.01, GraphPad Software, Inc., La Jolla, CA, USA), and MedCalc (version 11.4.2.0, MedCalc Software, Ostend, Belgium).

### Data availability

The datasets generated during and/or analysed during the current study are available from the corresponding author on reasonable request.

## Electronic supplementary material

Supplementary information
