# Supplementary material for: Blood T1* correction increases accuracy of extracellular volume measurements using 3T cardiovascular magnetic resonance: Comparison of T1 and T1* maps
Source: Sci Rep. 2018 Feb 20;8:3361. doi: 10.1038/s41598-018-21696-0 (PMC5820253; doi:10.1038/s41598-018-21696-0)
Supplement: Supplementary file 1 — Supplementary information [file 41598_2018_21696_MOESM1_ESM.pdf]

Blood T1* correction increases accuracy of extracellular volume measurements using 3T cardiovascular magnetic resonance: Comparison of T1 and T1* maps

Yongning Shang[1], Xiaochun Zhang[1*], Xiaoyue Zhou[2], Andreas Greiser[3], Zhengwei Zhou[4,5], Debiao Li[4], Jian Wang[1*]

1. Department of Radiology, Southwest Hospital, Third Military Medical University, Chongqing, China.
2. MR Collaboration, Siemens Healthcare Ltd., Shanghai, China.
3. Siemens Healthcare GmbH, Erlangen, Germany.
4. Biomedical Imaging Research Institute, Cedars-Sinai Medical Center, Los Angeles, California, USA.
5. Department of Bioengineering, University of California Los Angeles, Los Angeles, California, USA.

*Correspondence:

Jian Wang, Gaotanyan Street No.30, Shapingba district, Chongqing, China, Telephone, +8613883785811, Fax: +86-23-6546-3026, wangjian_811@yahoo.com;

Xiaochun Zhang, Gaotanyan Street No.30, Shapingba district, Chongqing, China, Telephone, +8615826056006, Fax, +86-23-6546-3026, zxcylxyr@163.com.

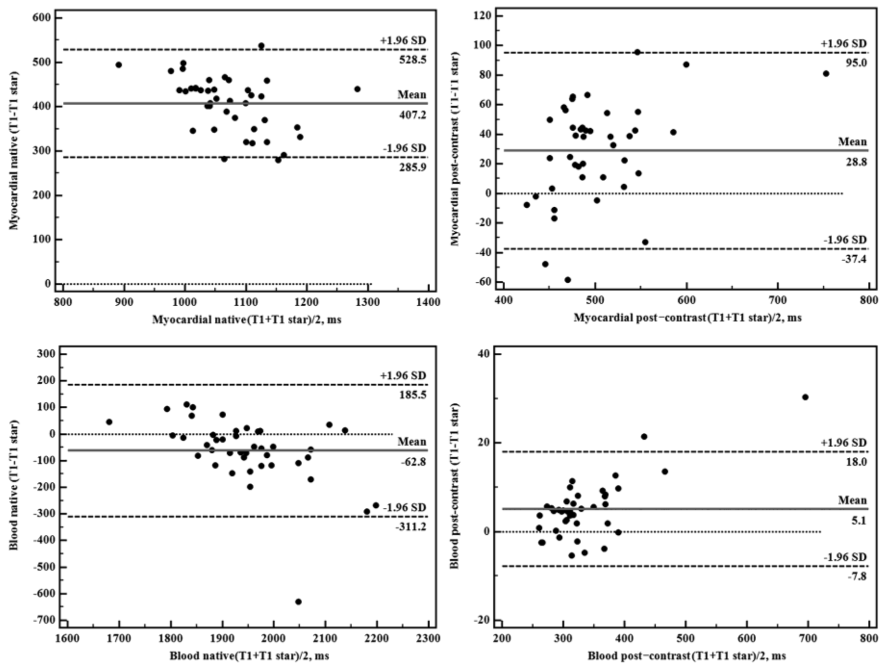

Supplementary Figure S1. Bland-Altman plots of T1 and T1* values. Bland-Altman plots show mean differences between T1 maps and T1* maps in global myocardium and blood T1 values.

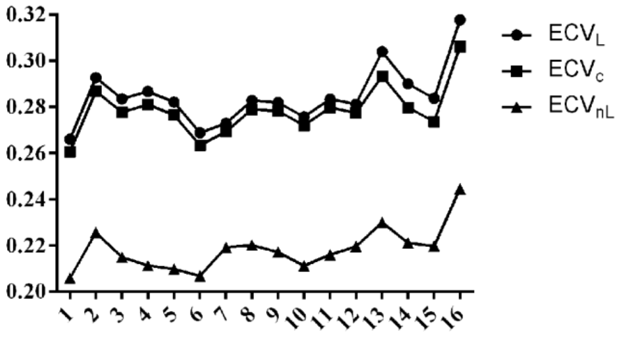

Supplementary Figure S2. Superimposed symbols with connecting line of ECV. Superimposed symbols with connecting line show the ECV_L, ECV_c, and ECV_{nL} of the American Heart Association 16-segment myocardium.

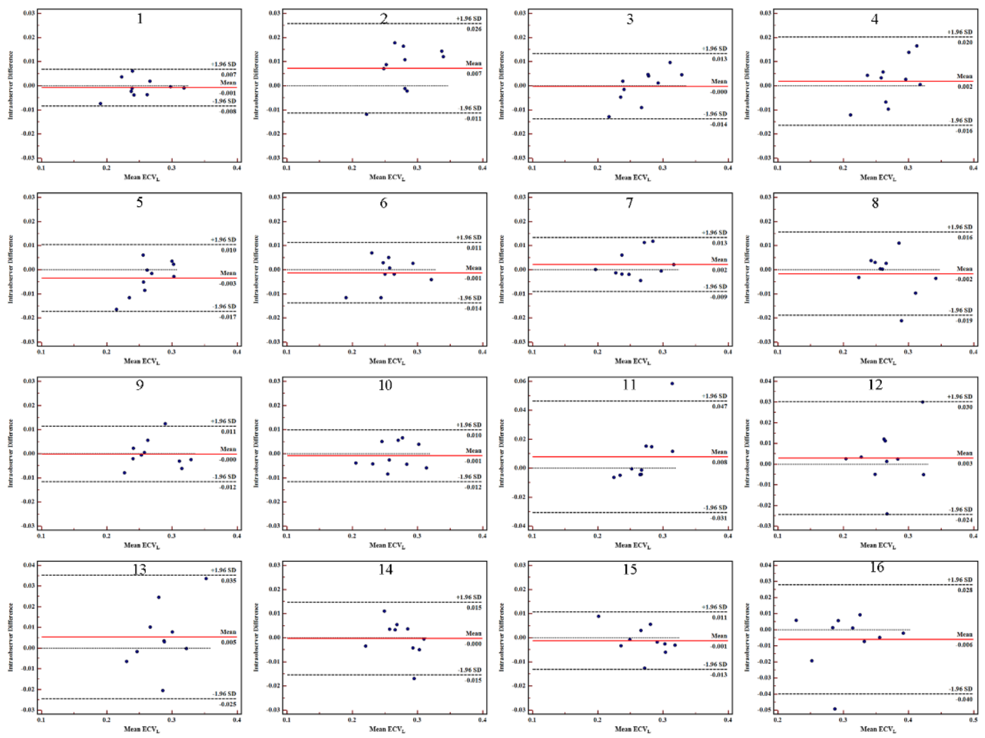

Supplementary Figure S3. Bland-Altman plots for intra-observer variability of ECV_L. Bland-Altman plots show intra-observer differences obtained for the ECV_L of the 16-segment myocardium.

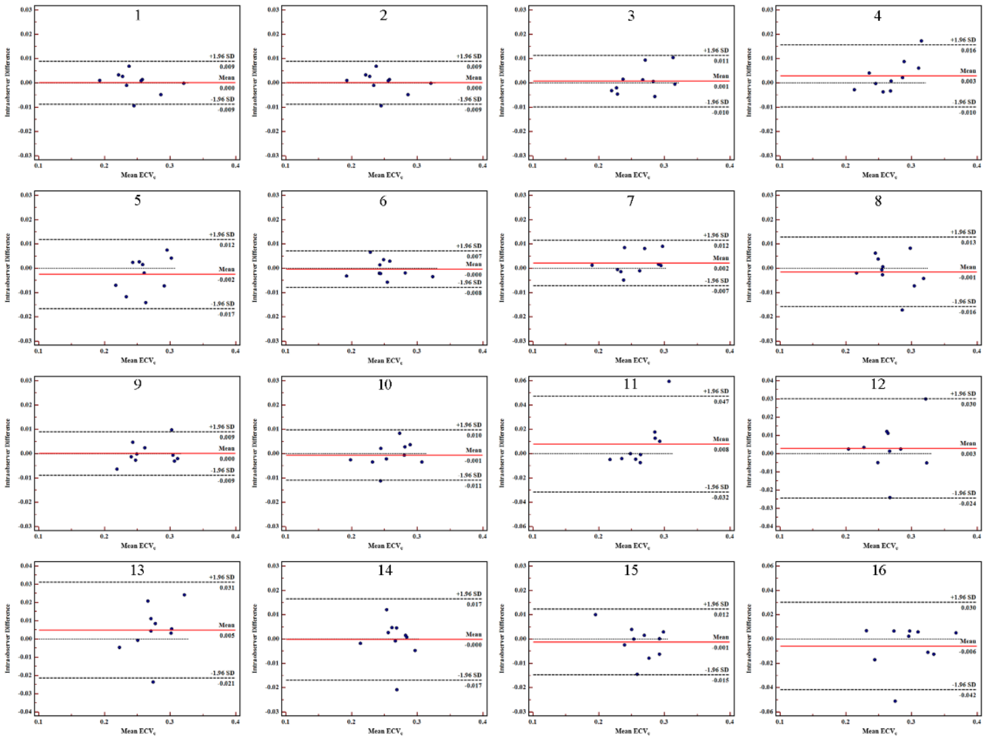

Supplementary Figure S4. Bland-Altman plots for intra-observer variability of ECV_C. Bland-Altman plots show intra-observer differences obtained for the ECV_C of the 16-segment myocardium.

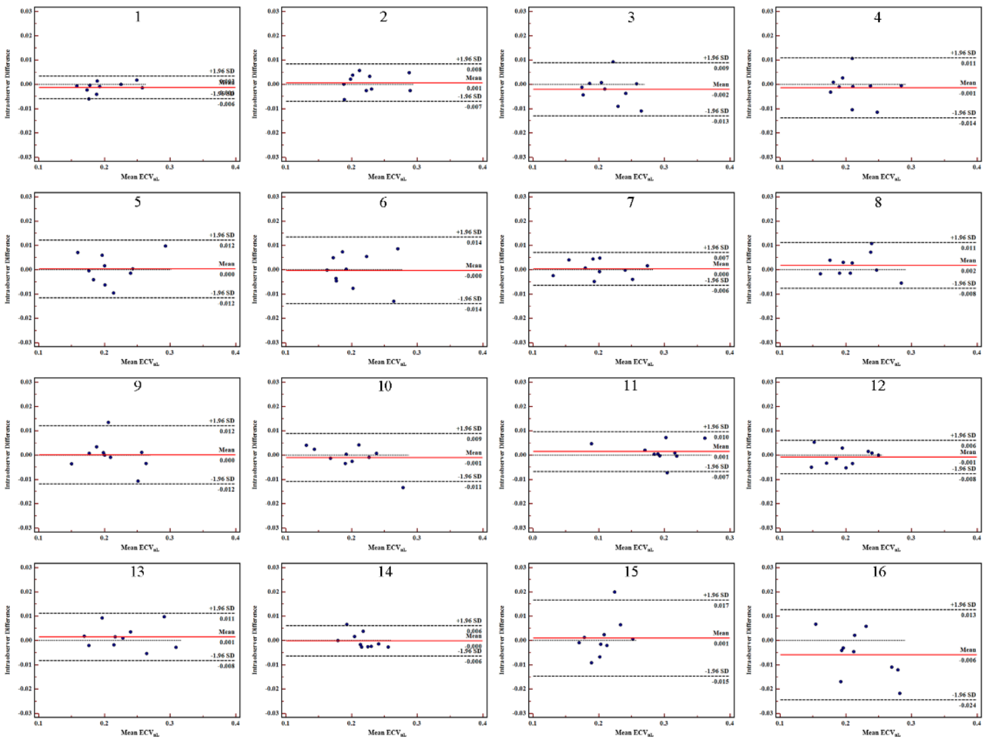

Supplementary Figure S5. Bland-Altman plots for inter-observer variability of ECV_{nL}. Bland-Altman plots show intra-observer differences obtained for the ECV_{nL} of the 16-segment myocardium.

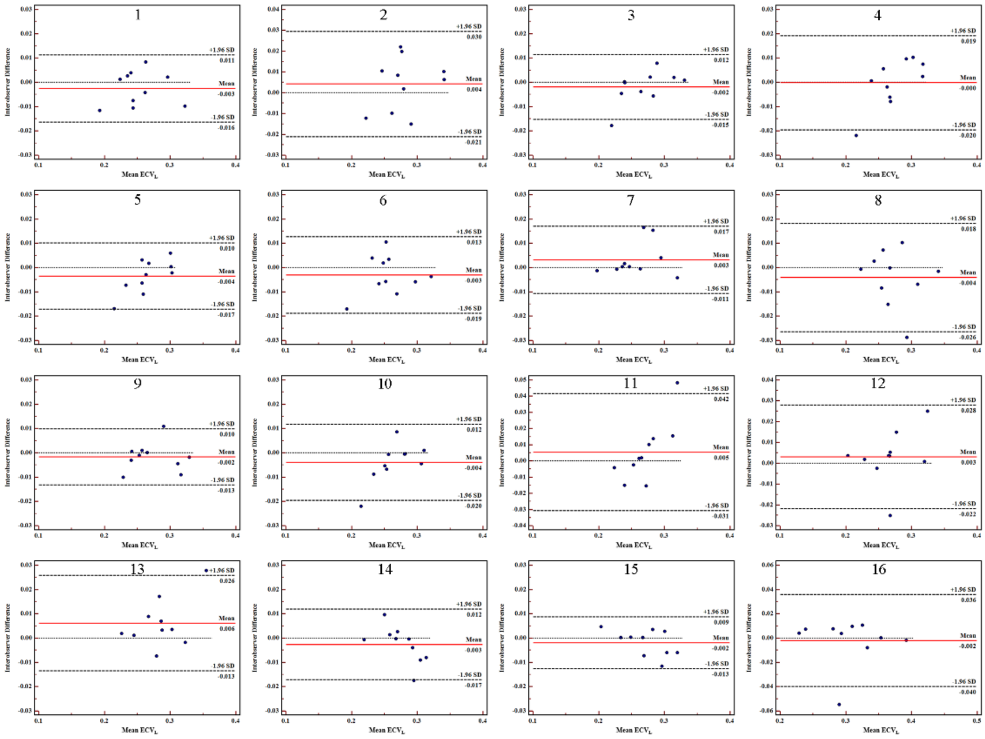

Supplementary Figure S6. Bland-Altman plots for inter-observer variability of ECV_{L}. Bland-Altman plots show inter-observer differences obtained for the ECV_{L} of the 16-segment myocardium.

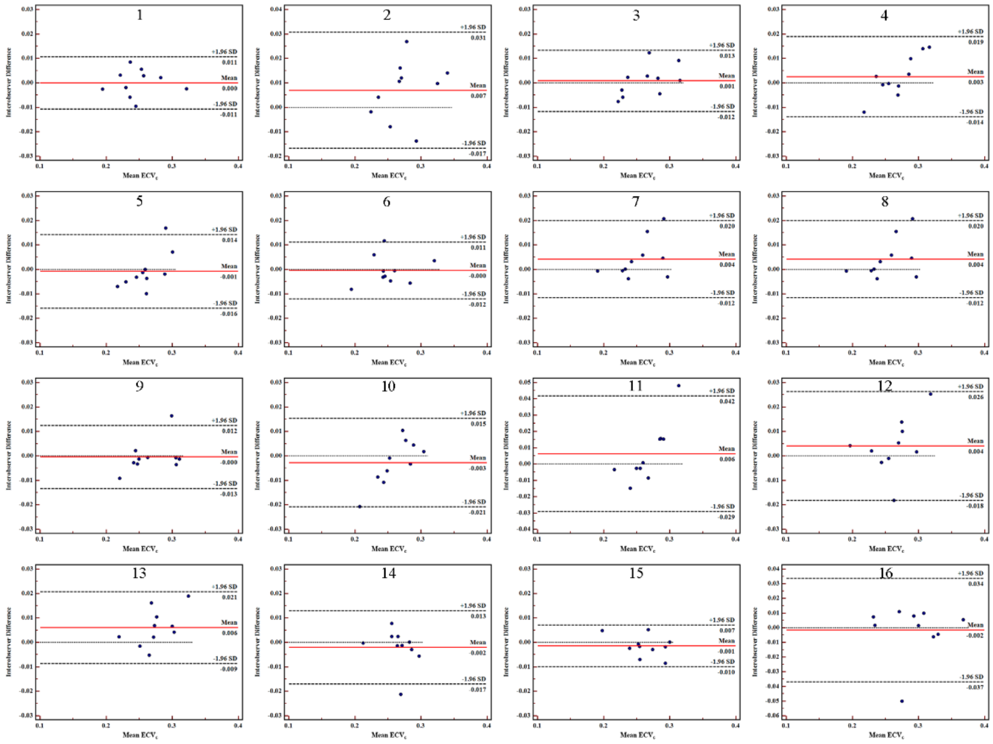

Supplementary Figure S7. Bland-Altman plots for inter-observer variability of ECV_c. Bland-Altman plots show inter-observer differences obtained for the ECV_c of the 16-segment myocardium.

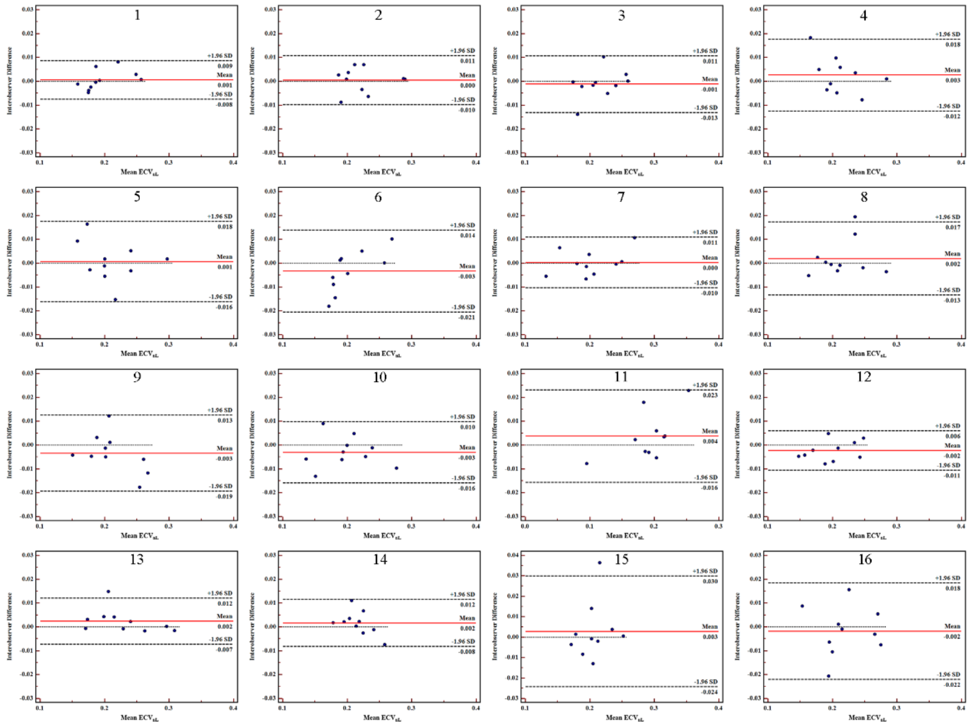

Supplementary Figure S8. Bland-Altman plots for inter-observer variability of ECV_{nL}. Bland-Altman plots show inter-observer differences obtained for the ECV_{nL} of the 16-segment myocardium.

Supplementary Table 1. Comparison LV myocardial ECV_L and ECV_{nL} all participants.

AHA Segments	ECV_L	ECV_{nL}	ECV_L - ECV_{nL}	(ECV_L - ECV_{nL})/ ECV_L, %	correlation coefficient	P
1	0.266 ±0.050	0.206 ±0.055	0.060	22.6	0.822*	<0.001
2	0.293 ±0.051	0.226 ±0.049	0.067	22.9	0.835*	<0.001
3	0.284 ±0.055	0.215 ±0.052	0.069	24.3	0.794*	<0.001
4	0.287 ±0.068	0.211 ±0.074	0.075	26.1	0.736*	<0.001
5	0.282 ±0.071	0.210 ±0.074	0.072	25.5	0.742*	<0.001
6	0.269 ±0.050	0.207 ±0.066	0.062	23.0	0.858*	<0.001
7	0.273 ±0.045	0.219 ±0.047	0.054	19.8	0.777*	<0.001
8	0.283 ±0.047	0.220 ±0.041	0.063	22.3	0.817*	<0.001
9	0.282 ±0.050	0.217 ±0.042	0.065	23.0	0.921*	<0.001
10	0.276 ±0.055	0.211 ±0.053	0.065	23.6	0.836*	<0.001
11	0.283 ±0.055	0.216 ±0.054	0.067	23.7	0.687*	<0.001
12	0.281 ±0.046	0.219 ±0.042	0.062	22.1	0.849*	<0.001
13	0.304 ±0.054	0.230 ±0.048	0.074	24.3	0.583*	<0.001
14	0.290 ±0.046	0.221 ±0.043	0.069	23.8	0.699*	<0.001
15	0.284 ±0.051	0.220 ±0.043	0.064	22.5	0.691*	<0.001
16	0.318 ±0.049	0.245 ±0.052	0.073	23.0	0.683*	<0.001
Global	0.285 ±0.049	0.218 ±0.044	0.066	23.2	0.896*	<0.001

correlation coefficient: linear regression between ECV and ECVs, *all p<0.001

Supplementary Table 2. Intra- and inter-observer reproducibility for ECV_L.

AHA Segments	Intra-observer		Inter-observer	
	Mean difference (95%CI), 10^{-3}	ICC (95%CI)	Mean difference (95%CI) , 10^{-3}	ICC (95%CI)
1	-1 (-8, 7)	0.994(0.977, 0.999)	-3 (-16, 11)	0.982(0.928, 0.995)
2	7 (-11, 26)	0.967(0.875, 0.992)	4 (-21, 30)	0.942(0.784, 0.985)
3	0 (-14, 13)	0.982(0.929, 0.995)	-2 (-15, 12)	0.983(0.932, 0.996)
4	2 (-16, 20)	0.963(0.860, 0.991)	0 (-20, 19)	0.957(0.837, 0.989)
5	-3 (-17, 10)	0.971(0.899, 0.993)	-4 (-17, 10)	0.972(0.892, 0.993)
6	-1 (-14, 11)	0.983(0.934, 0.996)	-3 (-19, 13)	0.974(0.899, 0.993)
7	2 (-9, 13)	0.987(0.951, 0.997)	3 (-11, 17)	0.981(0.925, 0.995)
8	-2 (-19, 16)	0.969(0.881, 0.992)	-4 (-26, 18)	0.946(0.801, 0.986)
9	0 (-12, 11)	0.987(0.947, 0.997)	-2 (-13, 10)	0.987(0.947, 0.997)
10	-1 (-12, 10)	0.986(0.944, 0.996)	-4 (-20, 12)	0.965(0.867, 0.991)
11	8 (-31, 47)	0.801(0.385, 0.947)	5 (-31, 42)	0.827(0.447, 0.954)
12	3 (-24, 30)	0.932(0.751, 0.983)	3 (-22, 28)	0.942(0.785, 0.985)
13	5 (-25, 35)	0.910(0.680, 0.977)	6 (-13, 26)	0.964(0.861, 0.991)
14	0 (-15, 15)	0.962(0.856, 0.990)	3 (-17, 12)	0.967(0.872, 0.992)
15	-1 (-13, 11)	0.986(0.944, 0.996)	-2 (-13, 9)	0.988(0.954, 0.997)
16	-6 (-40, 28)	0.938(0.773, 0.984)	-2 (-40, 36)	0.927(0.736, 0.981)

95%CI, 95% confidence Interval; ICC, intraclass correlation coefficient.

Supplementary Table 3. Intra- and inter-observer reproducibility for ECV_c.

AHA Segments	Intra-observer		Inter-observer	
	Mean difference (95%CI), 10^{-3}	ICC (95%CI)	Mean difference (95%CI) , 10^{-3}	ICC (95%CI)
1	0 (-9, 9)	0.992(0.968, 0.998)	0 (-11, 11)	0.988(0.952, 0.997)
2	0 (-9, 9)	0.958(0.839, 0.989)	7 (-17, 31)	0.946(0.798, 0.986)
3	0 (-14, 13)	0.988(0.954, 0.997)	-2 (-15, 12)	0.983(0.935, 0.996)
4	3 (-10, 16)	0.980(0.922, 0.995)	3 (-14, 19)	0.964(0.864, 0.991)
5	-2 (-17, 12)	0.966(0.869, 0.991)	-1 (-16, 14)	0.959(0.844, 0.990)
6	0 (-8, 7)	0.994(0.975, 0.998)	0 (-12, 11)	0.984(0.936, 0.996)
7	2 (-7, 12)	0.991(0.963, 0.998)	4 (-12, 20)	0.972(0.891, 0.993)
8	-1 (-16, 13)	0.974(0.899, 0.993)	4 (-12, 20)	0.972(0.891, 0.993)
9	0 (-9, 9)	0.991(0.965, 0.998)	0 (-13, 12)	0.980(0.924, 0.995)
10	-1 (-11, 10)	0.986(0.947, 0.997)	-3 (-21, 15)	0.951(0.817, 0.988)
11	8 (-32, 47)	0.774(0.322, 0.939)	6 (-29, 42)	0.817(0.422, 0.951)
12	3 (-24, 30)	0.923(0.721, 0.980)	4 (-18, 26)	0.947(0.801, 0.986)
13	5 (-21, 31)	0.892(0.626, 0.972)	6 (-9, 21)	0.967(0.875, 0.992)
14	0 (-17, 17)	0.931(0.747, 0.982)	-2 (-17, 13)	0.946(0.799, 0.986)
15	-1 (-15, 12)	0.976(0.905, 0.994)	-1 (-10, 7)	0.990(0.960, 0.997)
16	-6 (-42, 30)	0.906(0.668, 0.976)	-2 (-37, 34)	0.913(0.691, 0.978)

95%CI, 95% confidence Interval; ICC, intraclass correlation coefficient.

Supplementary Table 4. Intra- and inter-observer reproducibility for ECV_{nL}.

AHA Segments	Intra-observer		Inter-observer	
	Mean difference (95%CI), 10^{-3}	ICC (95%CI)	Mean difference (95%CI) , 10^{-3}	ICC (95%CI)
1	-1 (-6, 3)	0.998(0.999, 0.999)	1 (-8, 9)	0.992(0.969, 0.998)
2	1 (-7, 8)	0.994(0.978, 0.999)	0 (-10, 11)	0.990(0.960, 0.997)
3	-2 (-13, 9)	0.985(0.941, 0.996)	-1 (-13, 11)	0.980(0.922, 0.995)
4	-1 (-14, 11)	0.983(0.932, 0.996)	3 (-12, 18)	0.976(0.905, 0.994)
5	0 (-12, 12)	0.988(0.952, 0.997)	-1 (-16, 18)	0.978(0.914, 0.994)
6	0 (-14, 14)	0.983(0.934, 0.996)	3 (-21, 14)	0.968(0.879, 0.992)
7	0 (-6, 7)	0.997(0.988, 0.999)	0 (-10, 11)	0.992(0.967, 0.998)
8	2 (-8, 11)	0.992(0.967, 0.998)	2 (-13, 17)	0.977(0.910, 0.994)
9	0 (-12, 12)	0.986(0.946, 0.997)	3 (-19, 13)	0.977(0.911, 0.994)
10	-1 (-11, 9)	0.993(0.974, 0.998)	-3 (-16, 10)	0.988(0.953, 0.997)
11	1 (-7, 10)	0.995(0.982, 0.999)	4 (-16, 23)	0.971(0.890, 0.993)
12	1 (-8, 6)	0.995(0.981, 0.999)	-2 (-11, 6)	0.993(0.971, 0.998)
13	1 (-8, 11)	0.994(0.977, 0.999)	2 (-7, 12)	0.995(0.978, 0.999)
14	0 (-6, 6)	0.990(0.960, 0.997)	2 (-8, 12)	0.976(0.907, 0.994)
15	-1 (-15, 17)	0.950(0.813, 0.987)	3 (-24, 30)	0.851(0.512, 0.961)
16	-6 (-24, 13)	0.976(0.905, 0.994)	-2 (-22, 18)	0.966(0.871, 0.992)

95%CI, 95% confidence Interval; ICC, intraclass correlation coefficient.
